# Status of Hearing Loss and Its Related Factors among Drivers in Zahedan, South-Eastern Iran

**DOI:** 10.5539/gjhs.v8n8p66

**Published:** 2015-12-17

**Authors:** Hossein Ansari, Alireza Ansari-Moghaddam, Mahdi Mohammadi, Sayed Mehdi Tabatabaei, Babak Fazli, Mohammadreza Pishevare-Mofrad

**Affiliations:** 1 Health Promotion Research Center, Zahedan University of Medical Sciences, Zahedan, Iran

**Keywords:** automobile driving, hearing loss, noise

## Abstract

**Objective:**

This study aims to investigate loss of hearing among drivers in Zahedan, southeastern Iran.

**Patients and Methods:**

This study carried out on a total of 1836 drivers in Zahedan in 2013. Loss of hearing in both ears was measured at 250, 1000, 2000, 3000, 4000, 6000, and 8000 Hertz. The demographic variables, blood parameter and anthropometric data were recorded through interview and examinations. Data were analyzed in Stata.12 software using paired t-tests, McNemar test and Multiple Logistic Regression.

**Results:**

The mean age was 38.2±9.8 years. The highest mean hearing thresholds in the right and left ears were 25.7±9.1 and 27.7±9.1, respectively at 250 Hz. There was significant difference between left and right ears hearing threshold at all frequencies (P<0.001), and the highest difference occurred at 250 Hz. Hearing threshold in the left ear was greater than in the right ear at all frequencies. Hearing threshold was correlated to marital status, type of license, and vehicle, smoking, age, and driving history at all frequencies (P<0.01), and also significantly correlated to blood sugar and cholesterol levels at 250 and 500 Hz in both left and right ears (P<0.01).

**Conclusion:**

In conclusion, high levels of noise increase hearing threshold with greatest damage to the left ear. Therefore, drivers should be periodically examined for ear damage in accordance to variables affecting loss of hearing. Moreover, drivers must be educated about usage of appropriate ear-plugs during driving, especially for the left ear.

## 1. Introduction

Noise is a health-threatening factor, which can affect safety and efficiency of people in their workplace (Borchgrevink, 2003; Palmer et al., 2002). Through communication problems, and a lack of alertness and focus that will lead to stress and fatigue, noise can cause incidents and accidents and other occupational health/psychological consequences (Dalton et al., 2003; Kramer et al., 2002; Sprince et al., 2003), and this is often overlooked (da Silva et al., 2006; Picard et al., 2008). In terms of road accident mortality, Iran is among the world’s leading countries, so that according to statistics published by the Legal Medicine Organization, on average, 25,000 people lose their lives in road traffic accidents each year (Soori et al., 2009), and hearing loss can be a factor in creating road accidents (Ouis, 2001).

The most important complication associated with loud noise is loss of hearing, which can lead to masking noise and impaired communication, resulting in accidents. Thus, preventing hearing loss and screening those at risk can potentially reduce accidents. Currently, it is considered a major health issue and one of the 10 work-related diseases, and it is a major occupational disease in Europe (Nelson et al., 2005; Sulkowski et al., 2003). A study conducted in Poland showed that during 1998-2011, about 17.7% of occupational diseases belong to hearing loss (Szeszenia-Dąbrowska & Wilczyńska, 2013).

The noise is considered the third leading dangerous pollutant in major cities (Maleki et al., 2010), and road traffic is the most important cause of urban noise (Bluhm et al., 2004). This problem is further exacerbated by the growing number of vehicles on the urban road networks and their sluggish speed due to heavy traffic (Ouis, 2001). Thus, vehicles can be considered a moving source (traffic) of environmental noise and a source of occupational noise for drivers, which can affect their hearing.

Drivers are exposed to many physical and physiological stresses such as environmental noise, vibration, temperature fluctuations (due to opening and closing of door and working outside home in different seasons), ergonomic problems and safety risks such as accidents (Clark & Stansfeld, 2007; Jiao et al., 2004). Therefore, loss of hearing can be much more severe and have more complications in this group of people, compared to other occupations.

Many studies have been conducted in industrial environments on occupational hearing loss in Iran and around the world (Aliabadi et al., 2014; Leensen & Dreschler, 2015; Nomura et al., 2005), but only a few on loss of hearing in inner and intercity drivers. A study by Lopez et al. showed increased hearing threshold in 22% of drivers at 3000 to 6000 Hz, which increased further with aging (Lopes et al., 2012). Berjis et al. also reported that at 2000 Hz, left ear hearing threshold was significantly higher than that of the right ear among Heavy Goods Vehicle (HGV) drivers (Berjis et al., 2011). Additionally, some studies have found prevalence of hearing loss among drivers from 18.1% to 55.4% (Janghorbani et al., 2009, Santos & Castro Júnior, 2009).

Since in the Western countries, vehicles are much more advanced and create very little noise, only a few studies have been conducted on loss of hearing in drivers. However, a number of studies have been conducted in this area in developing countries. In a study in India, hearing loss in HGV drivers was 3 times more compared to taxi drivers (Merchant et al., 2000). Another study in Iran (Janghorbani et al., 2009) has reported the prevalence of bilateral noise-induced hearing loss about 18.1% and the prevalence rates had been higher in the left ear than the right ear.

On the other hand, there is no comprehensive study regarding drivers in South-East of Iran, Sistan & Bluchestan province. Furthermore, this province has been located at the border of Pakistan and Afghanistan where thousands of HV drivers transport goods from Iran to Pakistan or Afghanistan and vice versa. Therefore, study of hearing status and its related factors among drivers could be of utmost importance. Accordingly, the present study aimed to provide useful evidences on HV drivers’ hearing status and to identify groups at greater risk.

## 2. Material Studied

A total of 1836 inner and intercity drivers in Zahedan with a minimum of 5 years driving history were studied in terms of physical ear examination and audiometry in 2013. Study exclusion criteria were diseases of the ear, work history in noisy environments and subsequent loss of hearing. Participants included drivers with license class A and B (a classification of driving license in Iran), selected according to convenient sampling method. The demographic variables, history of hearing problems and work in other environments, blood parameter and anthropometric data were recorded in data registry forms through in-depth interviews and medical examinations and tests. Indeed, drivers were interviewed in predefined Health Center (Hanane) and a questionnaire on abovementioned information was completed by trained interviewers, for each subject. As we wanted to evaluate the relation between hearing loss and some blood parameters, so, they were asked to refer to the regional laboratory. The drivers additionally provided with an introduction letter for blood sampling (12-h overnight fasting). One day after the interview, a blood sample was taken from each driver at the laboratory and blood parameters were tested. The weight of the subjects was measured by standardized and reliable scale and height of them was measured in centimeter scale in standing position by height gauge.

After ear examination by an ear, nose and throat specialist, audiometry was performed by an audiometric clinician, and loss of hearing in both right and left ears was measured at 250, 500, 1000, 2000, 3000, 4000, 6000, and 8000 Hz, and recorded in audiogram sheets. The presence of a hearing loss was defined as a pure-tone average of threshold at 250, 500, 1000, 2000, 3000, 4000, 6000, and 8000 Hz greater than 25 dB of HL in the worse ear and the worse ear was chosen in order to include people with at least one affected ear (Rosenbaum & Stewart, 2004).

To ensure correct answers, study objective were explained for participants, and they were assured of confidentiality of data. Additionally, informed consent was obtained from all study participants. Data were analyzed using Paired t-test, McNemar and Chi-square test. Furthermore, a binary logistic regression was carried out with the Stata.12 software to clarify the predictors of HL. Normal distribution of data was confirmed using Kolmogrov-Smirnov test. P < 0.05 was considered significant.

## 3. Results

A total of 1836 inner and intercity drivers in Zahedan were studied, of whom, 385 drivers (21%) were younger than 30 years of age. 47 drivers (2.6%) were illiterate, and only 42 (2.3%) had university education. A total of 115 drivers (6.3%) were single, and 632 (34.4%) were HG drivers. 288 drivers (15.8%) (95% confidence: 8.8-3.2) were cigarette smokers.

Of all drivers, 438 (23.8%, CI95%: 22.1-25.5) had hearing loss in both ears, 77 (4.2%, CI95%: 2.8-5.6) had hearing loss in the right ear, and 187 (10.2%, CI95%: 8.9-12.1) had hearing loss in the left ear, with statistically significant differences (P=0.041). Permanent hearing loss was found in 45 drivers (2.4%) in the right ear, 69 (3.8%) in the left, and 28 (1.5%) in both ears, and at all frequencies, their hearing loss was greater than 25 Hz.

Frequency distribution of hearing loss in the right, left and both ears was found separately, and the relationship of hearing loss with demographic parameters and some blood factors was studied using logistic regression test (Table 1). The prevalence of hearing loss varied from 11.9% among drivers aged 20-29 years to 45.3% in those over 50 years of age. A significant relationship was found between age and hearing loss in the right and both ears as well (P<0.05). Moreover, there was significant relationship between hearing loss and type of vehicle (P = 0.042). In addition, passenger vehicle drivers had lower hearing loss than other drivers (Table 1).

**Table 1:** Frequency distribution of drivers’ hearing loss according to independent variables, together with odds ratio and confidence interval

Independent variables		No.	Hearing loss in the right ear		Hearing loss in the left ear		Hearing loss in both ears	
			n (%)	Age-Adjusted OR (95% CI)	n (%)	Age-Adjusted OR (95% CI)	n (%)	Age-Adjusted OR (95% CI)
Age (years)	20-29	385	10(2.6)	1	29(7.5)	1	46(11.9)	1
	30-39	638	19(3.0)	3.8(1.8-2.8)**	80(12.5)	1.2(0.72-2.2)	122(19.1)	6.1(4.1-9)**
	40-50	453	20(4.4)	3.3(1.8-2.6)**	41(9.1)	0.72(0.44-1.15)	130(28.7)	3.5(2.5-4.7)**
	Above 50	267	25(9.4)	2.2(1.2-4.1)**	25(9.4)	1.03(0.61-1.71)	121(45.3)	2.05(1.5-2.8)**
Education level	University	42	0(0)	1	4(9.5)	1	11(26.2)	1
	High school	529	21(4.0)	1.4(0.44-4.9)	45(8.5)	1.8(0.8-3.2)	296(24.4)	1.9(0.8-6.2)
	Junior high	1212	53(4.4)	1.6(0.47-5.7)	134(11.1)	1.3(0.6-6.2)	296(24.4)	1.3(0.7-5.6)
	Illiterate	47	3(6.6)	2.6(0.62-4.4)	3(6.4)	2.2(0.9-4.1)	26(55.3)	2.2(0.8-5.1)
Type of vehicle use	Passenger	166	13(7.8)	1	13(7.8)	1	24(14.5)	1
	Non-passenger	1671	64(3.8)	0.41(0.22-0.78)	174(10.4)	1.3(0.74-2.4)	414(24.8)	1.7(1.1-2.8)*
Type of vehicle	HGV	1204	46(3.8)	1	119(9.9)	1	286(23.8)	1
	Non-HGV	633	31(4.9)	0.66(0.4-1.07)	68(10.7)	0.94(0.67-1.3)	152(24.0)	0.84(0.66-1.08)
Marital status	Single	115	4(3.5)	1	9(7.8)	1	13(11.3)	1
	Married	1722	73(4.2)	0.69(0.22-2.1)	178(10.3)	1.07(0.55-2.2)	425(24.7)	1.2(0.65-2.3)
Smoking	Yes	288	17(5.9)	1	31(10.8)	1	106(36.8)	1
No	1549	60(3.9)	0.78(0.44-1.4)	156(10.1)	0.92(0.65-1.4)	332(21.4)	0.54(0.41-0.72)*
FBs	<110	1576	66(4.2)	1	164(10.4)	1	368(23.4)	1
	110-125	112	4(3.6)	1.1(0.43-2.2)	12(10.7)	1.4(0.34-3.4)	36(32.1)	0.66(0.42-1.04)
	>125	133	7(5.3)	1.36(0.38-4.8)	9(6.8)	0.63(0.25-1.50)	31(23.3)	1.15(0.55-3.3)
Cholesterol	<200	1096	50(4.6)	1	13(10.3)	1	248(22.6)	1
	200-240	455	18(4.0)	0.59(0.28-1.2)	54(11.9)	0.59(0.34-1.01)	106(23.3)	1.2(0.83-1.5)
	>240	270	9(3.3)	0.82(0.35-1.8)	18(6.7)	0.52(0.29-0.92)	83(30.7)	1.4(0.98-2.1)
TG	<200	1462	61(4.2)	1	148(10.1)	1	344(23.5)	1
	200-400	310	15(4.8)	0.59(0.28-1.2)	32(10.3)	1.1(0.42-2.8)	73(23.5)	1.5(0.82-3)
	>400	48	0(0)	0.82(0.35-1.8)	6(12.5)	1.1(0.41-3.1)	18(37.5)	1.7(0.89-3.5)
BMI	18.5-24.9	962	33(3.4)	1	100(10.4)	1	233(24.2)	1
	<18.5	101	5(5.0)	1.2(0.55-2.7)	6(5.9)	1.2(0.58-1.7)	24(23.8)	0.95(0.64-1.4)
	25-29.9	577	30(5.2)	1.17(0.22-2.3)	59(10.2)	1.7(0.66-4.6)	132(22.9)	1.27(0.42-2.43)
	>30	183	9(4.9)	1.2(0.35-2.8)	20(10.9)	1.05(0.59-1.81)	46(25.1)	1.15(0.7-1.5)
	Total	1873	77(4.2)		187(10.2)		438(23.8)	

* P<0.05,

** P<0.01; dds Ratio and 5%

CI calculated by binary logistic regression analysis.

Although HGV drivers more than non-HGV drivers, and illiterate drivers more than highly educated ones suffered loss of hearing, no significant relationship was found between loss of hearing and education level or type of vehicle (P>0.05). Similarly, the relationship of hearing loss with marital status was not significant (P>0.05). Hearing loss in smoker drivers was greater than in non-smokers, with a significant difference (P=0.038) (Table 1).

Although odds of hearing loss reduced with increasing blood sugar, cholesterol and triglyceride, none of these factors had a significant relationship with hearing loss (P>0.05). The odds of hearing loss increased with increasing BMI, but not significantly (P>0.05) (Table 1). It must be mentioned that the mismatch between the presented table and total sample is due to missing in some variables.

Frequency distribution of hearing loss and mean hearing threshold in both ears in drivers at different frequencies are shown in Table 2. Paired t-test showed a significant difference in hearing loss between left and right ears at 250, 500, 1000, 2000, 400, and 8000 Hz, and in every case. Hearing loss in the left ear was greater than in the right one, and the highest hearing loss frequency in both ears was at 250 Hz.

**Table 2:** Frequency distribution of hearing loss and mean hearing threshold in both ears in participants at different frequencies

Right ear	250Hz	500 Hz	1000 Hz	2000 Hz	3000 Hz	4000 Hz	6000 Hz	8000 Hz
Mean	25.7807	23.5106	16.6403	12.8451	11.6184	13.0235	13.5761	14.6541
SD	9.11157	8.48142	7.22941	7.26668	7.94837	9.11505	9.64528	9.58343
Minimum	.00	.00	.00	.00	.00	.00	.00	.00
Maximum	70.00	70.00	75.00	65.00	70.00	70.00	90.00	75.00
Hearing loss frequency N (%)	403(21.9)	281(15.3)	45(2.4)	35(1.9)	47(2.6)	75(4.1)	89(4.8)	93(5.1)
**Left ear**	250Hz	500 Hz	1000 Hz	2000 Hz	3000 Hz	4000 Hz	6000 Hz	8000 Hz
Mean	27.7400	23.8237	17.0797	13.1277	12.1043	13.4252	13.9334	15.1582
SD	9.16874	9.43199	8.62238	8.17705	8.45889	9.93787	9.94910	9.85579
Minimum	.00	.00	.00	.00	.00	.00	.00	.00
Maximum	90.00	90.00	90.00	60.00	85.00	90.00	75.00	70.00
Hearing loss frequency N (%)	548(29.8)	350(19.1)	88(4.8)	57(3.1)	55(3)	98(5.3)	103(5.6)	108(5.9)
P-value*	0.0001	0.0004	0.0001	0.003	0.2	0.009	0.14	0.12

* P value obtained using Paired t test.

**Figure 1:**
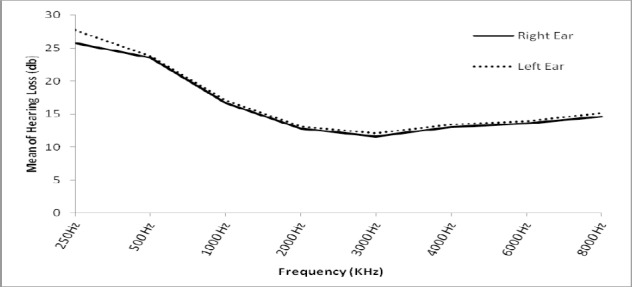
Trend of hearing loss at various frequencies for right and left ear

Figure 1 shows trend of hearing loss at various frequencies for right and left ear, indicating slightly greater hearing loss in the left ear than in the right one at all frequencies.

## 4. Discussion

The present study showed the prevalence of bilateral hearing loss in 23.8% of drivers, and about a quarter of drivers had hearing loss, which is higher than that previously reported in Iran (Janghorbani et al., 2009). There are few similar studies in Iran, and this is perhaps the first of its kind in the southeast.

Clearly, the prevalence of hearing loss depends on how it is defined, diagnostic methods, frequencies studied, age and gender, and socioeconomic status of drivers and study population in epidemiological studies, which limits comparison between studies. However, the previous studies conducted on the general population in Norway and USA (Agrawal et al., 2008; Borchgrevink et al., 2005) have reported the prevalence of hearing loss of 18% and 16.1%, respectively. Although the prevalence of hearing loss in general public is lower than in specific occupations, a study on phone operatives in Michigan (Stanbury et al., 2008) reported the prevalence of hearing loss 19%, which is less than that found in the present study. Generally, the high prevalence in the present study may be due to differences in definition of hearing loss, measuring technique, and study population. Another point is that although hearing status in different occupations is periodically examined, unfortunately this does not happen in the case of drivers. Thus, a lack of awareness or unavailability of proper services in the southeast of Iran can lead to drivers’ delayed visit to medical centers, resulting in increased hearing loss in this occupation.

Although hearing loss reduces with increasing blood sugar, cholesterol, and triglyceride, in multivariate analysis with controlled age, none of these parameters was significantly related to hearing loss. The odds of hearing loss increased with increasing BMI, but not significantly. This is somewhat in agreement with a previous study in Iran (Janghorbani et al., 2009). It appears that blood factors could be related to hearing loss in drivers but some studies conducted about the relation between blood factors and hearing loss, reported contradictory results (Daniel, 2007; Kaźmierczak & Doroszewska, 2000; Maia & Campos, 2005), which requires further investigation.

In the present study, haring loss in both ears increased with aging, which is in line with previous domestic and international studies (Janghorbani et al., 2009, Agrawal et al., 2008, Borchgrevink et al., 2005, Stanbury et al., 2008) in drivers and general populations. It seems, despite physiological and anatomic changes with aging; it is greater driving history and occupational exposure that cause hearing loss in drivers. As the design of devise has important role in voice emission (Bilski, 2013), so non-standard design of vehicles and automobile in Iran could be another factor that increase the hearing loss among Iranian drivers.

In this study, education level and marital status were not significantly related to hearing loss, but it was greater in passenger vehicle drivers than other drivers. Passenger vehicle drivers appear to spend more time driving and are more exposed to noise and stress, which adversely affects their hearing loss. Moreover, hearing loss was greater in smoker drivers than non-smokers. Although there are few studies on the effect of smoking on hearing loss, smokers appear to have higher stress and mental preoccupation. Generally, greater stress and noise have mutual effect on hearing loss. On the other hand, this result might be explained by the need to open the window when smoking, which in turn increases the exposure to noise.

This study showed greater hearing loss in the left ear than in the right one, and this agrees with studies in Iran (Berjis et al., 2011, Janghorbani et al., 2009) and worldwide (Kumar et al., 2005). According to tables 1 and 2, a similar situation is observed in subgroups of various parameters. It seems that the left ear is more exposed to noise through the vehicle window than the right one, and thus is more damaged. Use of an air-conditioner prevents leaving the window open, and thus prevents this situation.

Strong points in the present study included a large sample size, measuring demographics and blood factors, controlling confounding factors, and being the first study of its kind in the Southeast of Iran. Study limitations included being cross-sectional and not determining cause and effect relationship.

In conclusion, considering that proper hearing in drivers can have an important role in reducing preventing road accidents, it is essential that greater attention be paid to this occupation in terms of professional and occupational health, so that as well as preventing progress of hearing loss, safety of these and other people can be improved. It is recommended that drivers be periodically and regularly examined in terms of damage to the ear; and screening can be conducted according to parameters affecting hearing loss. Given the relationship between some blood factors and hearing loss, it is highly important to educate drivers about complications of hearing loss and use of appropriate ear-plugs during driving. Clearly, vehicle manufacturers can play a substantial role in reducing occupational hearing loss in drivers by standardization of production in terms of lower noise and air-conditioning to prevent opening of the window.
